# Estimating resilience of crop production systems: From theory to practice

**DOI:** 10.1016/j.scitotenv.2020.139378

**Published:** 2020-09-15

**Authors:** Matteo Zampieri, Christof J. Weissteiner, Bruna Grizzetti, Andrea Toreti, Maurits van den Berg, Frank Dentener

**Affiliations:** European Commission, Joint Research Centre (JRC), Ispra, Italy

**Keywords:** Agricultural production stability, Crop yield variability, Resilience indicator, Climate change, Extreme events, Climate adaptation, Crop diversity

## Abstract

Agricultural production systems are sensitive to weather and climate anomalies and extremes as well as to other environmental and socio-economic adverse events. An adequate evaluation of the resilience of such systems helps to assess food security and the capacity of society to cope with the effects of global warming and the associated increase of climate extremes.

Here, we propose and apply a simple indicator of resilience of annual crop production that can be estimated from crop production time series. First, we address the problem of quantifying resilience in a simplified theoretical framework, focusing on annual crops. This results in the proposal of an indicator, measured by the reciprocal of the squared coefficient of variance, which is proportional to the return period of the largest shocks that the crop production system can absorb, and which is consistent with the original ecological definition of resilience.

Subsequently, we show the sensitivity of the crop resilience indicator to the level of management of the crop production system, to the frequency of extreme events as well as to simplified socio-economic impacts of the production losses.

Finally, we demonstrate the practical applicability of the indicator using historical production data at national and sub-national levels for France. The results show that the value of the resilience indicator steeply increases with crop diversity until six crops are considered, and then levels off. The effect of diversity on production resilience is highest when crops are more diverse (i.e. as reflected in less well correlated production time series). In the case of France, the indicator reaches about 60% of the value that would be expected if all crop production time-series were uncorrelated.

## Highlights

• An easy-to-compute index to estimate crop production resilience is proposed.
• The proposed index quantifies the added value of crop diversity.
• Estimated resilience increases with higher agro-management levels.

## Graphical abstract

**Figure:**
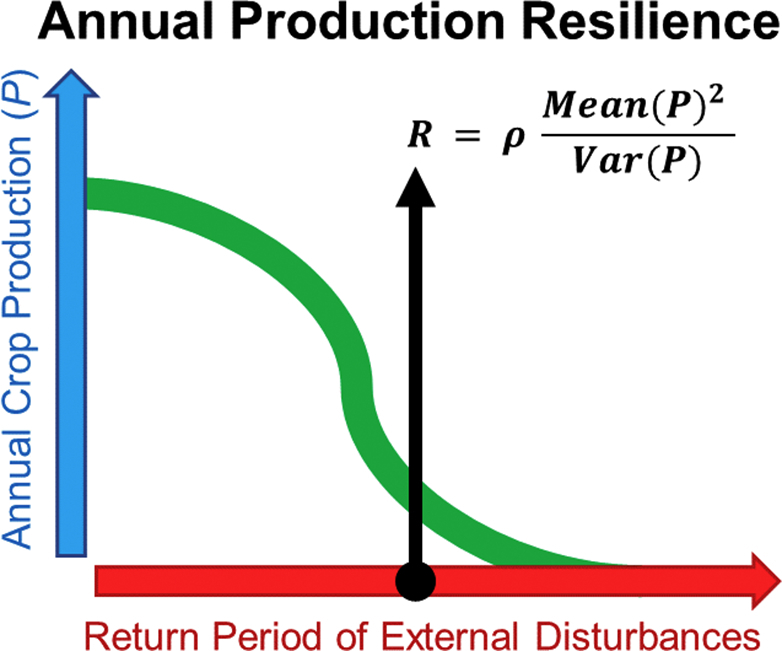

## 1 Introduction

The original meaning of the term resilience - first introduced in ecology – refers to the largest pressure that a system can cope with before changing its internal structure and losing its functioning capacity (Holling, 1973, Holling, 1996). Since then, the concept of resilience has been adopted and modified in other fields such as engineering and social sciences (Angeler and Allen, 2016; Angelo et al., 2013; Berbés-Blázquez et al., 2017; Brand and Jax, 2007; Folke, 2006; Quinlan et al., 2016). In the climate change context, for instance, a general definition of resilience is summarized by the IPCC (IPCC, 2014) as “the capacity of social, economic, and environmental systems to cope with a hazardous event or trend or disturbance, responding or reorganizing in ways that maintain their essential function, identity, and structure, while also maintaining the capacity for adaptation, learning, and transformation”.

Crops do indeed have a certain ability to resist and recover from unfavorable weather and climate events (e.g. heat waves, droughts, excessive rain, frost, etc.) and other environmental stresses that characterize local climate regimes. Crop production system resilience can be enhanced by increasing the level of adaptation and management, i.e. farm practices that target the most common stressors, by selecting resistant crop varieties, fine-tuning crop calendars, proper management of soil, water, nutrients, pests and diseases (Macholdt et al., 2019a, Macholdt et al., 2019b; Olesen et al., 2011; Peltonen-Sainio et al., 2009; Zhang et al., 2015).

However, extreme conditions related to late spring frost (Barranco et al., 2005; Eccel et al., 2009; Sweeney et al., 2013), heat stress and drought at flowering (Barnabàs et al., 2007; Descamps et al., 2018), failure of irrigation systems (Zampieri et al., 2018b), spread of pests and pathogens (Deutsch et al., 2018; Lamichhane et al., 2015; Savary et al., 2019), heavy precipitation or flooding that impede crop and seed development or harvesting activities (Lesk et al., 2016; Shaw et al., 2013; Zampieri et al., 2019b) can result in large production losses.

Current agricultural production systems are adapted to a range of local climate regimes, characterized by seasonal and inter-annual variations, which are subject to change due to global warming (Iizumi and Ramankutty, 2016; Lobell et al., 2011; Zampieri et al., 2015, Zampieri et al., 2018a, Zampieri et al., 2019a). However, with changing climate, extreme conditions such as heat waves and droughts can become more frequent and intense during the growing season. Therefore, agricultural systems that are considered resilient under current climatic conditions are likely to become less resilient, unless they adapt to such changes (Carr et al., 2016; Esper et al., 2017; Puma et al., 2015). Regardless of the level of agro-management, the most severe events (e.g. heat waves, droughts) will still be able to seriously damage crops (Gourdji et al., 2013; Schlenker and Roberts, 2009; Toreti et al., 2019; Zampieri et al., 2018b).

These issues motivate the need for a robust and simple method to quantify resilience of agricultural systems. Agricultural resilience and sustainability are generally assessed together in comprehensive frameworks accounting for socio-economic, biophysical, environmental and climatic indicators (Seekell et al., 2017; Zampieri et al., 2019b). Extensive literature reviews identified 30 relevant agro-ecosystem based sustainability indicators for climate resilient agriculture (Srinivasa Rao et al., 2018) and 15 different tools to assess resilience (Douxchamps et al., 2017). Notably, FAO developed a complex framework accounting for a large number of indicators (Russo and D'Errico, 2016).

Previous resilience analyses involved agricultural production indicators based on the mean and the inter-annual variability of crop production (Gil et al., 2017; Kahiluoto et al., 2019a; Khumairoh et al., 2018; Srinivasa Rao et al., 2018; Yang et al., 2019a, Yang et al., 2019b; Zimmerer and De Haan, 2017). The aim of this study is to contribute to the existing resilience assessment frameworks and to improve the understanding and the estimation of crop production system resilience from annual production time series. We propose an indicator that is directly linked to the ecological definition of resilience (Holling, 1973) applied to crop production systems.

The paper is organized as follows. First, we present the theoretical aspects of the annual crop production resilience indicator (*R*_*c*_), derived under some given strict assumptions. Then, we extend the indicator to more realistic non-stationary and diverse time series and we investigate the impact of crop diversity on the overall production system resilience. We apply the proposed indicator to two case studies: one based on simulated data for a single crop production system and one based on real world data of a diversified and evolving crop production system (i.e. the French commodity production system).

## 2 Material and methods

### 2.1 Annual crop production resilience indicator

#### 2.1.1 Homogeneous and stationary crop production system

First, we consider a simple agricultural production system with a single annual crop which grows on a spatial unit that is small enough to respond homogeneously to the pressures exerted by external forces such as weather and climatic events. We also assume that climate and agro-management do not change; that the annual crop production response to the forcing is stationary (i.e. time-independent); that the system bears no memory of the previous year conditions; and that any effect on this year's production has repercussion on the following year. Therefore, the mean and the variance of the production time series associated with this crop production system are constant. More realistic scenarios are discussed later in the paper.

We assume that there is a one-to-one (i.e. strictly monotonic) relationship between the amplitude of external shocks and their frequency of occurrence, this implies that the external forcing causing severe impacts is rarer than the one causing moderate disturbances. This condition is usually met, unless crops are grown in extremely unsuitable conditions. We also assume that there is a one-to-one relationship between the amplitude of external shocks and their impacts on the crop production system. These assumptions allow measuring the amplitude of the climate anomalies by their return periods of the impacts (T*), expressed in years. Using the return period allows accounting for shocks of different origins, such as different types of climate anomalies and other adverse events reducing crop production.

Thus, following the ecological definition of resilience (*R*_*e*_) as “the largest disturbance that a system can absorb before it loses its normal functioning” (Holling, 1973), we can define the resilience of the crop production system as the largest departure from the optimal conditions that the crop production system can sustain without losing its production capacity, measured by the return period (T*) of total production loss:

(1) $$R_{e}\equiv T*{}_{\mathrm{MAX}}$$

T*_MAX_ is equal to the reciprocal of the probability of crop failure (*F*) leading to total production loss:

(2) $$T*{}_{\mathrm{MAX}}=1/F.$$

Now, we consider the special case of annual crop production time series where the production values recorded at year *j* (*p*_*j*_) are either the potential (*p*_*j =*_ *P*) or zero (*p*_*j =*_ 0). This is equivalent to a crop production system that is optimally adapted to the local climate conditions (e.g. intensively managed systems with fully irrigated, fertilized and protected crops), developed to avoid the effects of moderate environmental stresses (T* < T*_MAX_), but still sensitive to extreme events such as floods, hailstorms or unseasonal frost whose return period exceeds a certain threshold (T* > T*_MAX_). We note that at large aggregated spatial scales (i.e. country or regional scales), total yield losses are very rarely observed, but they can occur more frequently and they can actually be observed at the field scale and for small spatial aggregations.

The mean and the variance of such time series are, respectively:

(3) $$\mu =P\;\left(1-F\right),$$

and

(4) $$\sigma^{2}=P^{2}\;\left(1-F\right)\;F.$$

For this simplified crop production system, it is possible to derive an indicator that is mathematically consistent with the ecological definition of resilience. We demonstrate it by combining Eqs. (3), (4). This allows obtaining the following relationships that eliminates *P* and relates the annual production mean and variance to total production loss probability:

(5) $$\mu^{2}/\sigma^{2}=\left(1-F\right)/F,$$

which is the inverse of the often used coefficient of variation, squared. For very rare events (F ≪ 1), the following approximation can be used: *(1* − *F)*/*F* ≃ *1*/*F.* The effects of this approximation, leading to a discrepancy of <10% for F < 0.1, will be discussed later in the numerical experiments and real data analysis.

Finally, using Eqs. (1), (2), we obtain the indicator for annual crop production resilience (*R*_*c*_, Zampieri et al., 2019):

(6) $$R_{c}\equiv \mu^{2}/\sigma^{2}≃R_{e},$$

as we wanted to demonstrate. In case a system is subject to frequent total production losses (e.g. F > 0.1), the relationship without approximation should be used (*R*_*c*_ *≡ μ*^*2*^/*σ*^*2*^ + 1).

We note that *R*_*c*_ depends on the ratio between the squared mean production and the variance, not solely on the individual terms. This information will be used later to define the algorithm for computing the resilience from non-stationary time series characterizing real production systems.

In addition, we also note that *R*_*c*_ increases with the mean annual production and decreases with the inter-annual variability. This basic property of the indicator is in agreement with the understanding of resilience as the minimization of the risk of production losses aspired by farmers (Macholdt et al., 2019c; Macholdt and Honermeier, 2017) and in line with other studies linking the concept of resilience and stability of crop production to the coefficient of variance (*σ*/*μ*) estimated on annual yield or production time series (Kahiluoto et al., 2019a; Renard and Tilman, 2019).

#### 2.1.2 Heterogeneous crop production system

In the previous section, we considered a single cropping system, exposed to a homogeneous external forcing and stationary conditions. In real-world conditions, however, crop production data are usually provided for larger heterogeneous spatial units with different crops and/or crop varieties and management conditions. We here account for this issue by considering different situations i.e. where crop responses to different climatic conditions are positively correlated, negatively correlated or uncorrelated.

The properties of R_c_ computed for the sum of crop productions under such conditions can be understood by induction considering two crops (*k* *=* *1,2*) and using the following statistical relationships linking the sum of two variables:

(7a) $$p_{j,\mathrm{TOT}}=p_{j,k=1}+p_{j,k=2}$$

(7b) $$\mu_{\mathrm{TOT}}=\mu_{1}+\mu_{2}$$

(7c) $${\sigma_{\mathrm{TOT}}}^{2}={\sigma_{1}}^{2}+{\sigma_{2}}^{2}+2\;\text{Covariance}\;\left(p_{k=1},p_{k=2}\right),$$

and then generalizing for an arbitrary number of crops. The production time series that are integrated may be related to different crops or different crop varieties, but also to the same crop under different climatic regimes and/or adaptation levels.

For simplicity, we consider an aggregated spatial unit composed of two homogeneous areas with two crops characterized by the same production mean and variance. The *R*_*C*_ indicator computed for the sum of these two production time series is characterized by the following particular cases depending on the cross-correlation of the production time series:

- if the crop production responses to a climate signal are perfectly correlated, the sum of the production is equal to twice the production of the single components; therefore, the mean and the standard deviation of the sum of the productions are doubled w.r.t. those of the individual production time series; consequently, *R*_*c*_ does not change w.r.t. the *R*_*c*_ computed from the individual production time series (*R*_*C,TOT*_ *= R*_*C*,k_).
- If the crop productions are perfectly anti-correlated, then the negative fluctuations of one spatial unit of crop production are fully balanced by positive fluctuations of the other crop's spatial unit, so that the resulting standard deviation of the summed production is zero and R_c_ is infinity: the crop production system has become perfectly resilient (*R*_*C,TOT*_ *= inf*).
- If the crop productions are uncorrelated, then the mean and variance of the sum are doubled w.r.t. those of the individual production time series (see Eq. (7c)), so that R_c_ of the total production will double as well (*R*_*C,TOT*_ *= 2*·*R*_*C*,k_).

In an agricultural system composed of an arbitrary number of *equivalent crops* (*k = 1, 2 … N*), i.e. characterized by the same production mean and variance but uncorrelated in time, the crop resilience indicator for the total production is equal to the crop resilience indicator of the individual crops multiplied by the number of crops (*R*_*C,TOT*_ *= N*·*R*_*C*,k_). This can be described as the *diversity theorem of the production resilience indicator*, in direct consequence of the *R*_*C*_ definition (Eq. (6)).

For uneven production levels, crops contribute proportionally to the overall crop system resilience. The same happens if the production time series are partially cross-correlated. Crop production time series with a much smaller *R*_*C*_ than for the primary crop can decrease the overall system resilience. The same reasoning holds in case time series of different crops are aggregated after converting the annual time series into nutritional values (e.g. energy or protein content) or into economic values for more meaningful comparability, depending on the specific application.

In order to quantify crop diversity, we adopt the inverse *Simpson dominance index* (Morris et al., 2014), defined as:

(8) $$S={\left({Ʃ}_{k}\;{\left(\mu_{k}/\mu_{\mathrm{TOT}}\right)}^{2}\right)}^{-1}$$

Comparing *S* to *R*_*C,TOT*_ allows establishing possible links between crop diversity and total resilience of diversified crop production systems as discussed in 2.2.2, 3.2.1. Compared to other popular diversity indexes such as the Shannon index, the Simpson dominance index attributes less weight to the time series with relatively small mean production that are less relevant for the computation of total resilience of the heterogeneous crop production system.

#### 2.1.3 Non-stationary production system

A technical difficulty for the estimation of resilience using real data is the presence of non-stationarity (trends) in the production time series due to technological development, improved management, expanding cropping areas, and other gradually varying factors that can affect both the mean and the variance (see e.g. Zampieri et al., 2017a, Zampieri et al., 2017b, Zampieri et al., 2019a).

Since the crop production resilience indicator definition (Eq. (6)) only depends on the ratio between mean production and variance, it is possible to normalize the production time series using the baseline trend defined as the smoothed time series, and to compute the resilience indicator for the normalized time series (Zampieri et al., 2020). This procedure assumes that system resilience is not changing when the standard deviation of the crop production anomalies varies proportionally to the baseline mean values and allows computing the resilience indicator using non-stationary data.

More specifically, we first compute the smoothed production time series using the LOESS procedure (Cleveland and Devlin, 1988) with span parameter set equal to 0.75 (Zampieri et al., 2017a, Zampieri et al., 2017b, Zampieri et al., 2019a, Zampieri et al., 2020), then we compute the normalized production anomalies and we derive the standard deviation:

(9) $${\underline{p}}_{j}=\text{loess}\;\left(p_{j}\right),$$

(10) $$\pi_{j}=\left(p_{i}-{\underline{p}}_{i}\right)/{\underline{p}}_{i}$$

(11) $$\sigma \prime =\mathrm{std}\;\left(\pi_{i}\right)$$

where the p_i_ represents the production values at year *j* of the time series under evaluation, π_j_ are the normalized anomalies with respect to the baseline values (i.e. the smoothed time series *p*_*j*_) and *σ*′ is the standard deviation of the normalized anomalies. The non-stationary crop production resilience indicator is simply given by the inverse squared standard deviation of the normalized anomalies:

(12) $$R\prime {}_{C}=1/\sigma \prime {}^{2}.$$

In case the production time series is stationary, R′_C_ is exactly equal to R_C_.

### 2.2 Simulated and observed crop production data

#### 2.2.1 Crop model formulation

We design a simple model to represent a homogeneous and stationary crop production system such as described in Section 2.1.1. It consists of different production impact functions that characterize the impacts of randomly generated annual external forcing. For a generic crop, the production is expressed as a function of the external forcing and the level of agro-management in terms of input intensity:

(13) $$p_{m,j}=P\cdot \left(1-D_{m}\;\left(T*{}_{j},T*{}_{\mathrm{MAX}}\right)\right),$$

where *D*_*m*_ is the normalized impact function (*0 < D*_*m*_ *< 1*). *D*_*m*_ depends on the return period of the external forcing occurring on year *j* (T*_j_), the return period of the maximum forcing that the crop system can absorb without losing completely the yield (T*_MAX_), and the agro-management level in terms of input intensity (*m*). In this simplified framework, the impact function *D*_*m*_ integrates the effects of farm practices such as soil-, water- and nutrient input intensity aiming at reducing the yield sensitivity to the most common local stressors (Yang et al., 2019a, Yang et al., 2019b).

The external forcing statistical properties as well as the response functions do not vary in time during the simulation. Several simulations are performed, with different frequencies of extreme events causing total yield loss (T*_MAX_) and several levels of adaptation to the common environmental stresses. Some simulations also include impacts lasting more than one cropping season to account – in a simplified manner - for socio-economic effects (defined later by Eq. (14)).

The crop model results in total yield loss every time when an external event with return period larger than T*_MAX_ occurs (T* > T*_MAX_). For less extreme events (T* < T*_MAX_) losses depend on the input intensity of the crop production system. This is accounted for by the different impact functions. Here we consider three cases

1) Linear impact function. This function represents – in a very simplified manner - crop production situations of low input intensity (*m* *=* *low*) as can be found in extensive agricultural areas and in developing countries. It is characterized by substantial production losses, even for very common environmental stresses and small departures from the average climatic conditions (T* ≪ T*_MAX_).
2) Stepwise impact function (Heaviside function). In contrast to (1), this function represents systems with high input intensity (*m* *=* *high*) that are sensitive only to extreme events, which happen at return periods larger than or equal to T*_MAX_. This is the situation that was considered to derive R_c_ (Eq. (6)) and can represent fully irrigated production systems with high level of fertilization; but where the crop is still heavily affected by specific climate extremes such as heat waves at flowering, spring frost and floods.
3) Intermediate polynomial function (third power). Compared to functions (1), (2), this function gives an intermediate production response to climatic variability, representing e.g. rainfed production systems in temperate humid regions with medium levels of fertiliser application (agro-management level, *m* *=* *mid*).

**Fig. 1:**
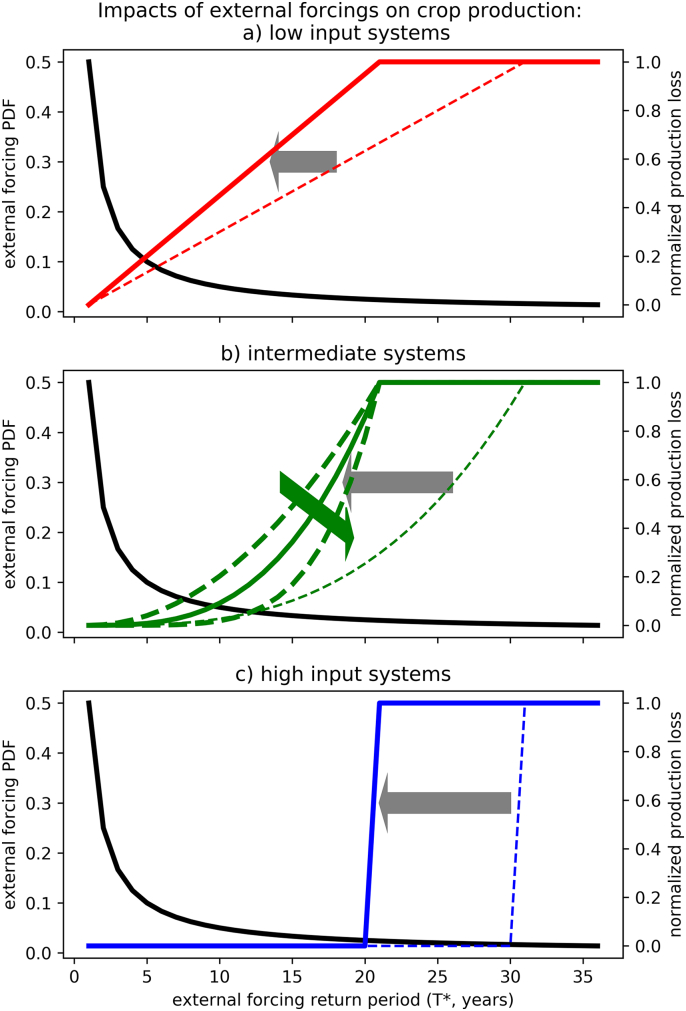
Hypothetical probability Density Function (pdf) of the external forcing (black line, left axis) plotted as a function of the return periods (T*). The chosen pdf belongs to the gamma family (shape = 1, scale = 2). Annual crop production impact functions (coloured lines, right axis) are shown for total production loss return periods: T*_MAX_ = 20 years (bold lines), and T*_MAX_ = 30 years (thin dashed lines). The impact functions for return periods smaller than T*_MAX_ represent three different types of crop production responses to external forcing: a) linear, b) polynomial (i.e. third power) and c) stepwise (Heaviside) in red, green and blue, respectively, representing low, moderate and high input intensities of the cropping system (m = low, mid, high). Bold dashed green lines in panel b represent the effect of adaptation that can be achieved by intensifying the inputs (green arrow), which tends to minimize the losses produced by the more common external forcings. The grey arrows represent the effects of climate change that increase the frequency of extreme events leading to total crop production loss for the different levels of input intensities.

Fig. 1 shows the statistical probability density function (*pdf*, black thick line) of the external forcing annual time series and six examples of impact functions for two values of T*_MAX_ and the above-mentioned three types of impact functions. The normal climate regime, with varying optimal or sub-optimal conditions for the crop, corresponds to relatively small values of T* compared to T*_MAX_. Extreme events that are detrimental for the crop, as reflected in large yield losses are associated with larger T*, or with T* > T*_MAX_ in the case of total yield loss. The impact functions in Fig. 1 are representative for two selected T*_MAX_ values (20 and 30 years), chosen as examples of different climate conditions.

Annual crop production time series can be also simulated accounting for cropping systems where the impacts of the external forcing persist for longer than one season, which could happen in real agricultural systems because of socio-economic processes such as, for instance, reduced investment capacity after a ‘bad’. This case can be simulated in our simplified framework using the same impact functions as described above, but summing to the loss of year *j*, for instance, half of the loss of the previous year (*j-1*):

(14) $$D\prime {}_{m,j}=D_{m,j}+D_{m,j-1}/2$$

Different impacts and longer timescales could be considered as well.

#### 2.2.2 Real world data: the agricultural commodities production system in France

We provide an example of application of the proposed crop production resilience indicator for heterogeneous and non-stationary crop production systems. We focus on France and we use FAOSTAT data (http://www.fao.org/faostat/) and AGRESTE data (agreste.agriculture.gouv.fr/) available at the country level (NUTS-0) and at the departments level (NUTS-3), respectively.

The main crops considered in the FAOSTAT and AGRESTE data sets are wheat, maize, barley, rapeseed, sugar beet, sunflower seed, potatoes, triticale and oats. In the AGRESTE dataset, wheat is separated between soft and durum. Soft wheat, durum wheat and barley are further distinguished between winter and spring varieties.

In order to compare and sum the productions of different commodities, we use mass-to-energy conversion tables from USDA and FAO. The value we used are, in kilocalories per kilogram, 3310 for wheat, 3650 for maize, 3540 for barley, 4940 for rapeseed, 430 for sugar beet, 5840 for sunflower seed, 770 for potatoes, 3360 for triticale and 3890 for oats.

The effect of crop diversity on resilience is then analysed summing progressively the production time series of the different commodities (previously sorted in decreasing order of mean caloric annual production) and computing the resilience indicator on the resulting time series using Eqs. (9), (10), (11), (12) at the national level and for each spatial aggregation. The total value for the resilience indicator of the system is then compared to the reciprocal Simpson diversity index (Eq. (8)).

If the sum of the production time series is not computable for at least 25 years and for at least the first five commodities (due to missing data), the spatial aggregation is categorized as not suitable to estimate the diversity-resilience relationship and excluded from the overall analysis. This criterion leaves us with 92 out of 101 departments (according to the 2016 definition of the spatial aggregations).

Since the reliability of the estimates for the individual departments is affected by the limited length of the time series (Kahiluoto et al., 2019b; Piepho, 2019; Snowdon et al., 2019), we estimate the sampling error of *R*_*C*_ by using Monte Carlo simulations of synthetic production time series, assuming a Gaussian distribution of production around the mean, which is often the case for production data aggregated over administrative units (Zampieri et al., 2019a; Zampieri et al., 2017a, Zampieri et al., 2017b) and also suggested by our considerations on heterogeneous crop production systems (Section 2.2) because of the *Central Limit Theorem* in probability theory. The accuracy of the indicator is quantified in Section 3.2.2 for different samples varying number of years and for different *σ*/*μ* ratios.

## 3 Results

### 3.1 Crop model experiments: impacts of climate change and adaptation on crop production resilience

In this section we compute the annual production resilience indicator (Eq. (6)) from time series generated by the simple crop model described in Section 2.2.1 for different frequencies of extreme events, representing different climates, and different impact functions representing the different levels of agro-management for a homogeneous crop production system.

**Fig. 2:**
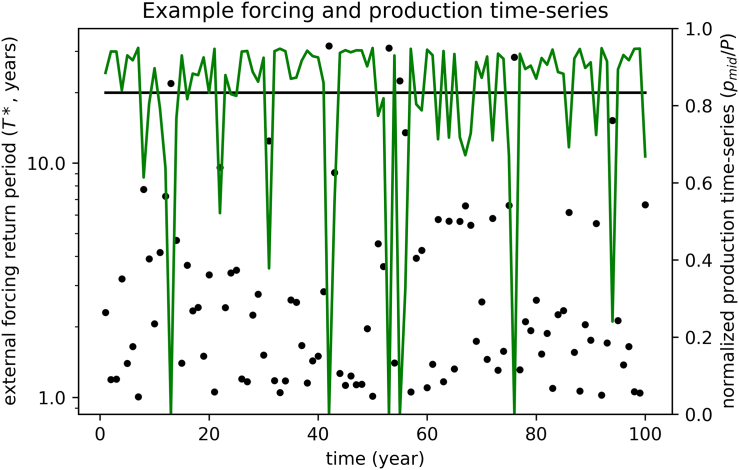
Example time series of external forcing (dots, expressed as the corresponding return periods T*) and production (green lines, normalized) from an idealized model simulation obtained with polynomial impact function (corresponding to intermediate input intensity, m = mid) and total yield loss return period of 20 years. The horizontal line represents the critical threshold of the forcing that produces total yield loss.

Fig. 2 shows a randomly generated time series of external forcing (black dots) following the prescribed probability density function *pdf* (black line in Fig. 1) and the corresponding annual crop production time series resulting from the polynomial impact function (green line in Fig. 1). In this simple model simulation, the critical threshold of the return period is set to 20 years (T*_MAX_ *= 20*, horizontal line in Fig. 2), implying that total production loss occurs on average every 20 years (it occurs 4 times in the random realization shown in Fig. 2). The annual crop production resilience indicator computed for the production time series plotted in Fig. 2 is equal to 11.2.

**Fig. 3:**
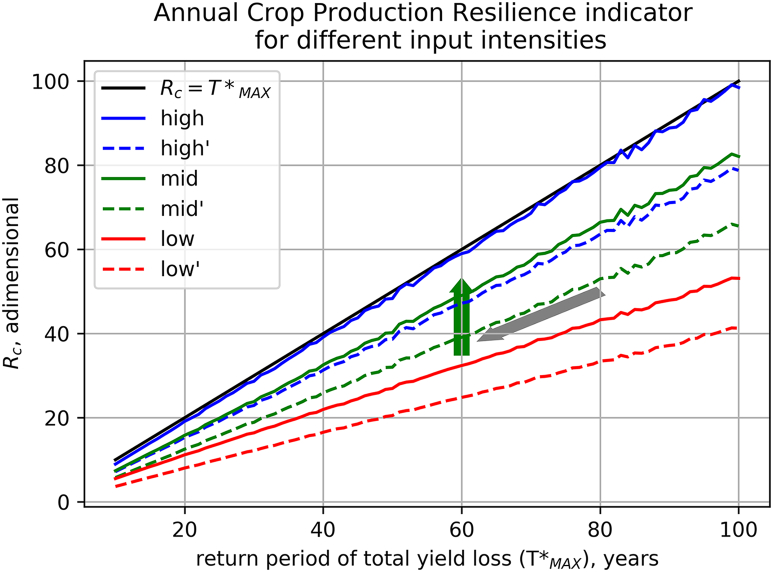
Annual crop production resilience indicator (R_c_) computed for return periods ranging from 10 to 100 years and for different impact functions: stepwise function representing high input intensity (m = high, blue lines), polynomial function for moderate input intensity (m = mid, green lines), linear function for low input intensity (m = low, red lines). Dashed lines show the estimated R_c_ for cropping systems influenced by the external forcing triggering effects propagating in time as given by Eq. (14) (high′, mid′, low′, see text for explanation). As in Fig. 1, the arrows show the direction of the effects of climate change (grey) and of inputs intensification (green) on R_c_.

Fig. 3 summarizes the results of similar experiments computed for all the crop impact functions depicted in Fig. 1 and for a wide range of return periods of total production loss (T*_MAX_ = 10 to 100 years), also including impacts lasting longer than one season.

Fig. 3 shows the *R*_*c*_ indicator computed from annual crop production time series for cropping systems characterized by different return periods of total yield loss (T*_MAX_) and different management intensities. In general, the *R*_*c*_ indicator is proportional to T*_MAX_ and decreases for lower input intensities and for impacts lasting longer than a single growing season, in agreement with common sense understanding of resilience. In all the considered cases, near-linear relationships between *R*_*c*_ and return period of total yield loss (T*_MAX_) are found. The departures from linearity, because of the approximation leading to the definition of *R*_*c*_ (Eqs. (5), (6)) for short return periods (i.e. high values of *F*), are too small to be visible in Fig. 3. The proportionality factor of the relationship decreases in crop production systems with lower management intensity and bearing memory of the climate impact of the previous year. As expected, the R_c_ indicator computed for the system with a stepwise impact function, corresponding with intensively managed systems (*high*, blue line) is (almost) identical to T*_MAX_. Considering longer recovery times from the impacts exerted by the external forcing (*high′*) leads to a lower R_C_ of about 30. For the polynomial impact functions considered, about the same R_c_ is recorded for intermediate level of management and with no time-propagation effects (*mid*). In case of high sensitivity to moderate environmental stresses (i.e. linear impact function corresponding with low level of management), R_c_ is about half the value of the intensively managed agricultural system (*high*) at the same T*_MAX_, and further decreases in case socio-economic impacts are considered as well.

In this simplified framework, the effects of climate change and of the form of adaptation to climate change consisting of increasing input intensity can be easily understood as shifting the crop production system, in the resilience space represented in Fig. 3, along the directions represented by the grey and green arrows, respectively. In absolute terms, the resilience of crop production systems with low input intensity is less affected by changes in the frequency of extreme events compared to the more developed systems because the production variability is largely determined by the impacts of smaller perturbations from the optimal climate conditions. In absolute terms, the impacts of climate change on crop production resilience will be easier to detect in more intensive cropping systems. However, the relative effects are the same.

### 3.2 Real-world case study: effects of crop diversity on resilience and accuracy of the estimation

#### 3.2.1 Effects of crop diversity on resilience, the example of France

**Fig. 4:**
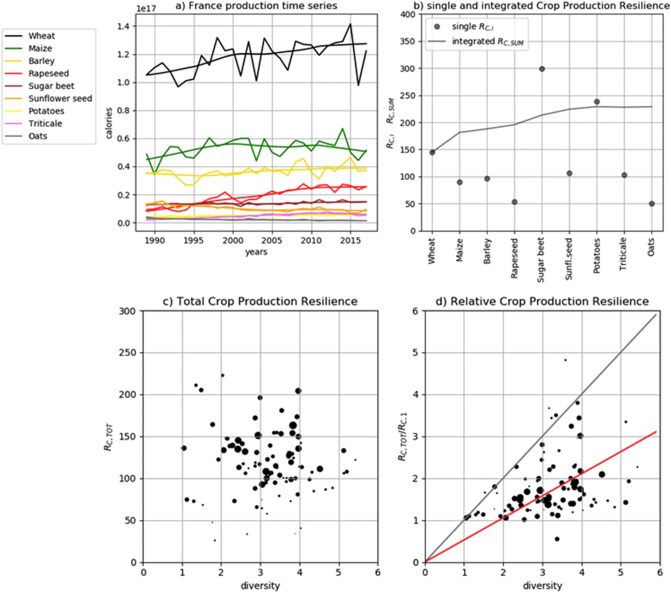
a) Annual caloric production time series recorded for the main French commodities at the national scale (NUTS-0) since 1989. b) Crop production resilience indicator computed for the individual time series (dots) and for the time series obtained by summing progressively each of the crop production time series (line), in descending order of total share. c) Total crop resilience at department (NUTS-3) levels versus crop diversity. The size of the markers is proportional to the caloric production means for each spatial unit. d) As 4c, bit for the relative crop resilience, obtained by dividing the total resilience by the resilience of the primary crop (i.e the one with the largest caloric production) in the same department. The red line shows the weighted linear interpolation of the plotted values.

Fig. 4a and b shows the crop production resilience and diversity estimated for France using FAOSTAT data. Wheat is the most important crop for the caloric production, followed by maize, barley and the other commodities. The crop diversity index computed for the French commodity system using the Simpson index (Eq. (8)) is equal to 3.6.

The smoothed lines in Fig. 4a correspond to the non-linear fitting used to normalize the time series before computing the annual crop production resilience indicator (*R*_*C*_) of the individual crop production time series. For wheat, we note the exceptional production loss occurred in 2016 (Ben-Ari et al., 2018). The 2016 anomaly is less pronounced for the other main crops. Synchronized climate impacts can be observed as a result of the 1994 and 2003 heat waves as well (Zampieri et al., 2009). On the other hand, some example of compensation can be observed in other years, such as in 2001 and 2007.

Fig. 4b shows the crop resilience indicator of the individual crop production time series at the national level and the crop resilience indicator computed for the time series obtained adding step-wise the individual crop production time series in decreasing order of mean calories production.

The estimated annual production resilience (*R*_*C*_) of wheat — the primary French crop — is about 145 while the estimated *R*_*C*_ of maize — the next crop in decreasing order of energy production— is 90. Nevertheless, when the caloric production values of the two crops are summed up, the combined *R*_*C*_ is larger, reaching about 180. Considering barley as well, characterized by similar resilience of maize, the integrated resilience reached almost 190. Integrated resilience still increases adding rape seed, albeit characterized by lower resilience than the previous crops. Integrated resilience starts to level off when sugar beet and sunflower seeds productions are added, with negligible contribution to the total annual production resilience from other crops.

Fig. 4c shows the total crop resilience computed for each departments of France (NUTS-3 level) using AGRESTE data and plotted against crop diversity as calculated by the inverse Simpson index (Eq. (8)). Each department is characterized by different levels of annual production resilience, reflecting the diverse agro-climatic conditions of France (Ceglar et al., 2016) that mask the effects of local crop diversity on total resilience. Annual production resilience values of about ten or less, which could possibly question the validity of the assumption leading to the definition of the resilience indicator (Eqs. (5), (6)), are never found in the French production data.

Fig. 4d shows the same result of Fig. 4c, but for the relative resilience, obtained by dividing the total resilience of each department by the resilience of the most import crop locally. This procedure filters out the regional differences isolating the gain of resilience attributable to crop diversity. In this case, we observe a clear tendency of increasing annual crop production resilience with increasing crop diversity. In Fig. 4d, relative total resilience is distributed below the y = x line, which would be the expected theoretical values if the time series of the individual crops were uncorrelated. A weighted linear regression model for this data estimates a ratio of 0.52 between the relative crop resilience and crop diversity, which is statistically significant at the 99.9% level.

#### 3.2.2 Uncertainty associated with the annual production resilience indicator

Table 1 shows the results of the Monte Carlo procedure that we have used to quantify the effects of limited duration time series of the relative uncertainty of the annual production resilience indicator estimation. The accuracy of R_C_ estimation highly depends on the standard deviation. Therefore, long-time series are preferred for a more reliable estimation of R_C_. From Table 1, we also note that it is more difficult to estimate crop resilience for low resilience systems (i.e. small R_c_), but this is much less important than having time series of sufficient length. Comparing the resilience indicator from different location can be a strategy to increase the sample size in order to derive meaningful relationships such as the one between resilience and diversity (Kahiluoto et al., 2019a; Renard and Tilman, 2019), as we also showed in the previous Section 3.2.1.

**Table 1:** Relative uncertainty (in %) of R_c_ computation for crop production time series with different number of years (n) and different σ/μ ratios.

σ_Rc_/R_C_	*σ*/*μ* = 5% (R_c_ = 400)	*σ*/*μ* = 10% (R_c_ = 100)	*σ*/*μ* = 20% (R_c_ = 25)	*σ*/*μ* = 30% (R_c_ = 11.1)	*σ*/*μ* = 50% (R _c_ = 4)
n = 10	63%	64%	65%	67%	73%
n = 20	37%	38%	38%	39%	44%
n = 30	28%	29%	29%	30%	34%
n = 40	24%	24%	25%	26%	29%
n = 50	21%	21%	22%	23%	26%
n = 100	15%	15%	15%	16%	18%

## 4 Discussion

We emphasize that the annual production resilience indicator proposed is derived from the ecological definition of resilience applied to production systems. This and other interesting properties distinguish the annual production resilience indicator from other stability indicators such as the coefficient of variance. The reciprocal of the coefficient of variance is a well-known indicator for stability (e.g. Renard and Tilman, 2019), sometimes associated to resilience (Kahiluoto et al., 2019a). We have shown that the production resilience indicator bears clearer and more intuitive properties compared to the reciprocal of the coefficient of variance. In the cases considered in this paper, the production indicator is inversely proportional to the frequency of extreme external forcing, and linearly correlated with the crop production system diversity and with the spatial variability of the climatic impacts to crop production.

The application of the proposed resilience indicator, to both simulated and observed data has several important implications. For the simple crop production systems discussed in this paper, we have found that climate change can affect more strongly the resilience of crop production systems under intensive management (because of the larger proportionality factor) with respect to extensive systems. This corroborates the finding that agronomic improvements favouring high yields are not necessarily decreasing their sensitivity to climate variability (Lobell et al., 2014; Twomlow et al., 2008).

The case study for France that we addressed as an example of application of the proposed indicator strongly points to the benefit of crop diversification to resilience at country level (Renard and Tilman, 2019) as well as at subnational levels. Further research will extend this result at the European level that we plan to do. However, when considering multiple cropping systems, it is also important to take into account the potential of different adaptation strategies with respect to raising inputs intensity of agro-management level. Highly adapted systems can react to climate change by growing diverse suitable crops and conserving biodiversity (Bahri et al., 2019).

Our method and results are generally consistent with recent literature on crop production stability (Kahiluoto et al., 2019a; Renard and Tilman, 2019; Yang et al., 2019a, Yang et al., 2019b). However, some studies raised the issues of data requirement to measure resilience reliably and of the lack of theoretical ground of resilience indicators (Piepho, 2019; Snowdon et al., 2019). Our study is addressing both questions. Specifically, we also noted that the main difficulty in the estimation of the relationships between resilience and diversity is related to data availability and quality, which depend on both the target area and the spatial scale. While crop diversity can be easily computed from the production means of the individual commodities, just one missing value for one crop in one year in principle prevents computing the total (multi-crop) production for that year, shortening the time-series available to derive total production resilience, and limiting the accuracy of the resilience estimation. However, we demonstrated that quantitative considerations on the effects of crop diversity on the resilience of crop production systems are possible using the available observations, adding evidence to the recent debate on this topic (Kahiluoto et al., 2019b).

## 5 Conclusions

This study provides a theoretical framework for addressing quantitative aspects of resilience and sustainability of real crop production systems using models and observed data for different world regions and at the global scale in the context of climate change (Zampieri et al., 2019, Zampieri et al., 2020). While most adaptation studies often neglect the importance of inter-annual crop production variability (Challinor et al., 2014), our theoretical findings demonstrate how it can be accounted in order to assess resilience. If the stability of the production time series is measured by the reciprocal of the coefficient of variance of the production time series, we have demonstrated that the resilience indicator proposed is the square of the stability (Zampieri et al., 2019) and we provided a definition that can be applied to non-stationary time series characterizing agricultural production (Zampieri et al., 2020).

When applied to real data, the production resilience indicator can capture the overall integrated effects of agronomical, socio-economic, political and global environmental factors such as agricultural development (i.e. adaptation and management), climate variability, and socio-economic factors. Therefore, it could be a corner stone in the collection of resilience/sustainability indicators adopted in the holistic socio-economical resilience assessments methods.

The computation of the production resilience indicator is relatively straightforward but also has limitations, in particular concerning the sample size (i.e. the length of the available production time series to compute the index). For a crop production system with at least 30 years of production data, the uncertainty of the resilience estimation is <30%. Larger sampling errors are associated to shorter time series and for crop systems displaying larger inter-annual fluctuations compared to the production mean.

The sampling issue can be overcome by considering longer datasets, which can be generated by crop models, or by comparing and aggregating data over larger spatial units (Zampieri et al., 2019, Zampieri et al., 2020). Simulation model experiments and an example case study for France point to an important role of adaptation and management, and confirm the importance of crop diversification to enhance production resilience.

More specifically, we have demonstrated that the crop resilience indicator computed for spatially aggregated or diversified crop production systems is generally larger than for homogeneous systems. In case of an idealized system composed of multiple crops with uncorrelated production time series, total crop resilience is predicted to increase proportionally to the number of equivalent crops. Our analysis of crop resilience and diversity relationships performed on the French agricultural production system suggests a proportionality factor of about 0.5 (instead of 1), which is attributed to the correlations among the production time series (e.g. for wheat and barley).

In addition to crop diversity, we claim that strategies aiming at adapting both the crop distribution and the varieties to the changing climate conditions, instead of intensifying the inputs, can increase the crop production resilience by possibly reducing the overall effects of increasing extreme events, while respecting sustainability boundaries.

## CRediT authorship contribution statement

**Matteo Zampieri:**Conceptualization, Methodology, Formal analysis, Visualization, Data curation, Writing - original draft, Writing - review & editing.**Christof J. Weissteiner:**Conceptualization, Data curation, Writing - original draft, Writing - review & editing.**Bruna Grizzetti:**Writing - original draft, Writing - review & editing.**Andrea Toreti:**Writing - original draft, Writing - review & editing.**Maurits van den Berg:**Writing - review & editing.**Frank Dentener:**Writing - review & editing.

## Declaration of competing interest

The authors declare that they have no known competing financial interests or personal relationships that could have appeared to influence the work reported in this paper.
